# Improving APT Systems’ Performance in Air via Impedance Matching and 3D-Printed Clamp

**DOI:** 10.3390/s23115347

**Published:** 2023-06-05

**Authors:** Liu Liu, Waleed Abdulla

**Affiliations:** Department of Electrical, Computer and Software Engineering, The University of Auckland, Auckland 1010, New Zealand

**Keywords:** APT system in air, piezoelectric transducer, impedance matching, clamp

## Abstract

This paper presents a study on improving the performance of the acoustic piezoelectric transducer system in air, as the low acoustic impedance of air leads to suboptimal system performance. Impedance matching techniques can enhance the acoustic power transfer (APT) system’s performance in air. This study integrates an impedance matching circuit into the Mason circuit and investigates the impact of fixed constraints on the piezoelectric transducer’s sound pressure and output voltage. Additionally, this paper proposes a novel equilateral triangular peripheral clamp that is entirely 3D-printable and cost-effective. This study analyses the peripheral clamp’s impedance and distance characteristics and confirms its effectiveness through consistent experimental and simulation results. The findings of this study can aid researchers and practitioners in various fields that employ APT systems to improve their performance in air.

## 1. Introduction

Wireless power transfer (WPT) is an innovative technology representing a crucial advancement in the domain of power transfer [1,2,3]. This method can potentially revolutionize traditional energy utilization patterns in various applications, such as portable electronic devices, implanted medical devices, integrated circuits, electric vehicles (EVs), and so on. Unlike traditional wires, the WPT technique transmits energy from the power source to the target through the air. Near-field and far-field transmissions constitute this new form of energy. Near-field utilizes the inductive coupling effect of nonradiative electromagnetic fields, which includes both inductive and capacitive mechanisms. Far-field WPT can be achieved using acoustic, optical, and microwave energy carriers.

**Figure 1:**
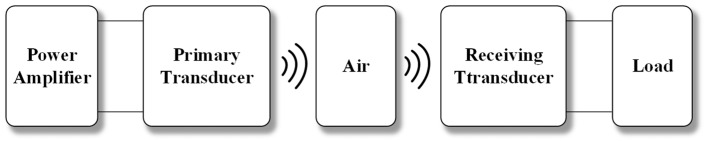
Acoustic power transfer system.

Acoustic power transfer (APT) is a newer wireless power transfer (WPT) form. Energy transmission is accomplished using sound waves as intermediate energy carriers [4,5]. As shown in Figure 1, piezoelectric transducers transform electrical energy into vibrations and continuously propagate them as pressure waves. A receiver, situated at a specific point along the wave’s path, converts the electricity back into sound energy. There are three types of medium in which it can travel: gaseous, fluid, and solid. Compared with other wireless powering techniques, such as inductive power transfer and midrange RF power transmission, acoustic power transfer (APT) has some advantages, such as lower tissue absorption, a shorter wavelength that enables more miniature transducers [6], and an increase in power intensity threshold for safe operation. The main advantage of APT is non-reliance on electromagnetic fields to transmit energy. It can be used in environments with strong electromagnetic fields.

The performance of an acoustic power transfer (APT) system depends on several factors, including the material properties of the piezoelectric transducers [7] and the size and geometry of these transducers. For example, [8] optimized the converter to reduce the system’s losses. Another crucial factor that significantly affects the system’s performance is impedance matching. While APT technology is commonly used with solid and fluid mediums, its usage in air is rare. This is primarily because the acoustic impedance of piezoelectric transducers differs significantly between air and other mediums, causing a significant mismatch, as illustrated in Table 1. The acoustic impedance of piezoelectric transducers is similar to that of solids and liquids.

In contrast, it is considerably lower in air, resulting in substantial losses when using APT systems in an airborne setting. Therefore, studying the energy transfer of APT in air is of great importance. Due to its minimal impact on biological tissues and the environment, APT can be applied to medical detection and monitoring inside the human body, and it can also offer new technological support for developing smart homes, IoT, and other fields. This paper aims to enhance APT systems’ performance in air by implementing impedance matching.

Impedance matching enhances system performance and stability by minimizing reflection and loss during the energy transfer process. Generally, two impedance matching methods are used for APT systems to facilitate energy transfer in air. One traditional approach addresses abrupt impedance changes near the interface of the propagation medium and the piezoelectric transducer by inserting a reflective layer between them. Typically, this layer consists of specialized materials. Ref. [9] proposed a method to quantify the impact of acoustic impedance mismatches on performance and demonstrated that optimizing the thickness of intermediary layers can resolve these impedance issues. The optimization of transducer performance involves proposing fabrication methods and modelling for the matching layers, as described in [10]. Other special materials such as polyether sulfone and polystyrene foam can also be placed between the transducer and the propagation medium to adjust the impedance [11,12,13,14,15,16,17,18]. The ideal intermediate matching layer in an APT system should ideally have an impedance equal to the geometric mean of the propagation medium and the transducer. However, materials with extremely low impedance, which are required for this purpose, are typically not available in pure form or as a single phase. Moreover, using multiple layers of material matching layers increases attenuation.

Alternatively, instead of traditional matching techniques, an acoustic meta-surface can be employed. Acoustic meta-surfaces consist of ultrathin 2D building blocks that allow for manipulation of sound phase. These meta-surfaces find applications in acoustic lenses [19], sound diffusers [20], acoustic holograms [21], as well as impedance-matched surfaces and wavefront engineering. For instance, a simple meta-surface composed of thin membranes and tiny air cavities provides an efficient impedance matching surface for water-to-air communication [22]. Ref. [23] proposed non-local waterborne acoustic meta-surfaces (WAM) with highly non-local features for efficient underwater acoustic control, taking into account fluid–solid interactions (FSIs) to manipulate underwater sound with higher efficiency. However, this method increases the volume of the system.

Finding an impedance matching method that satisfies both low attenuation and no increase in volume is challenging. To overcome this problem, a new impedance matching method for APT systems in air has been developed. The study found that the fixed position of the piezoelectric transducer plays a crucial role in impedance matching, although there are limited studies on this aspect. Typically, the back of the piezoelectric transducer is fixed. To develop a contact model, a comprehensive impedance analysis is performed on systems with structural connections and contact interfaces, using the principle of equivalence between mechanical and electrical characteristics. In this paper, the piezoelectric transducer is fixed on the back [24]. Ref. [25] proposed a self-detached APT system, which can be easily installed and removed in harsh environments. However, it is still fixed on the back of the piezoelectric transducer. However, different positions of the fixed piezoelectric transducer result in different vibration patterns, which can impact system performance. Therefore, studying the fixed position of the piezoelectric transducer is particularly important. In this paper, the effect of impedance matching on the efficiency of the APT system is examined using a one-dimensional circuit model, demonstrating its effectiveness. The influence of the piezoelectric transducer’s clamp position on vibration patterns and sound pressure level is simulated using COMSOL. Additionally, a novel detachable peripheral clamp is designed using 3D printing, significantly reducing the cost. The output voltage of the APT system is compared using both the novel peripheral clamp and the traditional clamp, revealing an increase of nearly 200 mV with the new peripheral clamp, highlighting its effectiveness.

## 2. Matching Theory of Acoustic Impedance

**Figure 2:**
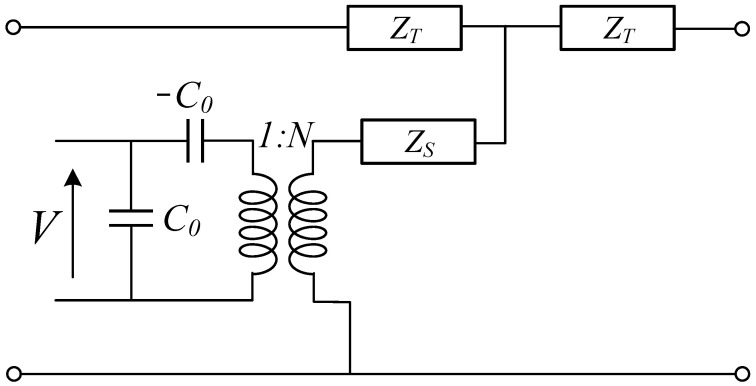
Mason’s one-dimensional equivalent circuit.

Various modern technologies employ PTs for signal transmission, making PT a critical area of research. However, there are many theories for studying PT, including classical theory [26], Rayleigh’s theory [27], Bishop’s theory [28], and finite element model simulations [29]. Among them, equivalent circuits are one of the most frequently used calculation methods for PTs, which utilize mechanical properties to analogize circuit parameters. There are several types of circuit models, but the Mason circuit is one of the most popular. As a valuable tool for solving one-dimensional problems, network theory was utilized by Mason to create a more precise equivalent circuit based on this approach. This circuit divides the piezoelectric transducer into three ports, one electrical and two acoustic, using an ideal electromechanical transformer, as shown in Figure 2 [30,31]. The electrical port represented is made up of merely two capacitors, whose values are equal to *C*_0_, but the electrodes are in opposite directions. *N* represents the electromechanical coupling, and the impedances (*Z_T_*, *Z_S_*) stand for the mechanical properties. The other parameters of a Mason circuit are shown in Table 2. Wu presented a novel equivalent circuit model for typical UWPT systems. They chose the T network as the electro–ultrasound–electric channel description, which significantly improved the calculation of circuit characteristics [32]. Mason equivalent circuits have also developed a slew of other formulations, including analogue networks [33], KLM circuits, and systems models [34]. However, there are only a few papers that investigate impedance mismatch using these equivalent models.

### 2.1. Impedance Matching Equivalent Circuit

**Figure 3:**
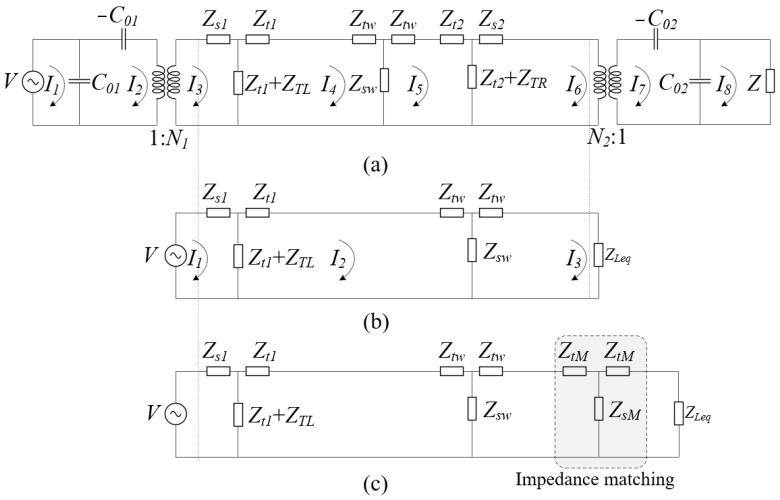
(**a**) Mason’s one-dimensional equivalent circuit; (**b**) simplified diagram of Mason’s one-dimensional equivalent circuit; (**c**) Mason’s one-dimensional equivalent circuit with impedance matching.

To verify that impedance matching can optimize system performance and that efficiency is an important expression of system performance, we chose efficiency to verify that impedance matching is effective for improving system performance. The method of this research is to calculate the partial derivative of the system efficiency and the impedance value of the matching layer in order to determine the value of impedance matching, which is used to improve the system performance. However, deriving formulas using the original Mason model would result in highly complex equations, impeding observation and practical applications. Therefore, the Mason one-dimensional equivalent circuit was first simplified, as shown in Figure 3. Figure 3a is a original Mason circuit, and Figure 3b is a simplified Mason circuit. Equation (1) shows the system efficiency equation derived from the simplified Mason circuit. However, the expansion of the efficiency formula is still very complicated; some parameters with small resistance in the circuit are ignored. The ultimate simplified efficiency formula is displayed in Equation (5), which can be derived by simplifying Equation (1). Equation (2) is the equivalent impedance parameter. Equations (3) and (4) are the currents *I*_1_ and *I*_3_ of the simplified Mason circuit in Figure 3b.

(1) $$\eta_{1}=\frac{Re\left[V_{out}\;I_{3}^{*}\right]}{Re\left[V\;I_{1}^{*}\right]}$$

(2) $$\begin{array}{l} Z_{11}=Z_{s1}+Z_{t1}+Z_{tL}\\ Z_{12}=Z_{t1}+Z_{tL}\\ Z_{22}=Z_{t1}+Z_{tL}+Z_{t1}+Z_{tw}+Z_{sw}\\ Z_{21}=Z_{t1}+Z_{tL}\\ Z_{23}=Z_{sw}\\ Z_{33}=Z_{tw}+Z_{sw}+Z_{leq}\\ Z_{32}=Z_{sw} \end{array}$$

(3) $$I_{1}=\frac{-\left(V\cdot \left(Z_{22}\cdot Z_{33}-Z_{23}\cdot Z_{32}\right)\right)}{\left(Z_{11}\cdot Z_{23}\cdot Z_{32}-Z_{11}\cdot Z_{22}\cdot Z_{33}+Z_{12}\cdot Z_{21}\cdot Z_{33}\right)}$$

(4) $$I_{3}=\frac{-\left(V\cdot \left(Z_{21}\cdot Z_{32}\right)\right)}{\left(Z_{11}\cdot Z_{23}\cdot Z_{32}-Z_{11}\cdot Z_{22}\cdot Z_{33}+Z_{12}\cdot Z_{21}\cdot Z_{33}\right)}$$

(5) $$\eta_{2}=\frac{R_{Leq}}{R_{Leq}+\left|{\frac{Z_{33}}{Z_{32}}|}^{2}R_{t1}+\right|{\frac{Z_{22}Z_{33}-Z_{23}Z_{32}}{Z_{21}Z_{32}}|}^{2}R_{s1}}$$

(6) $$\eta_{1}\approx \eta_{2}$$

**Figure 4:**
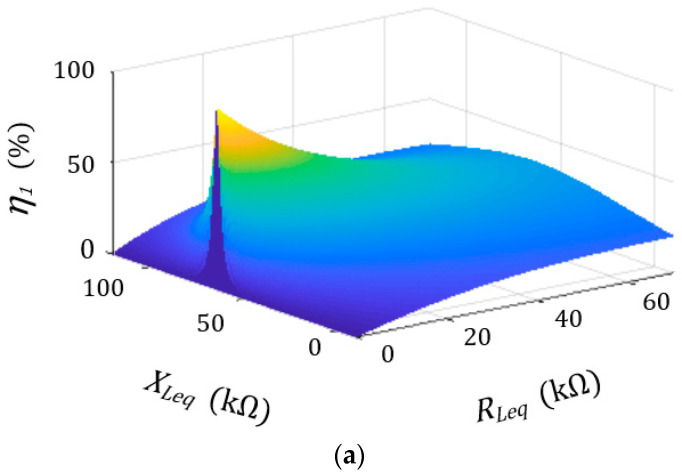

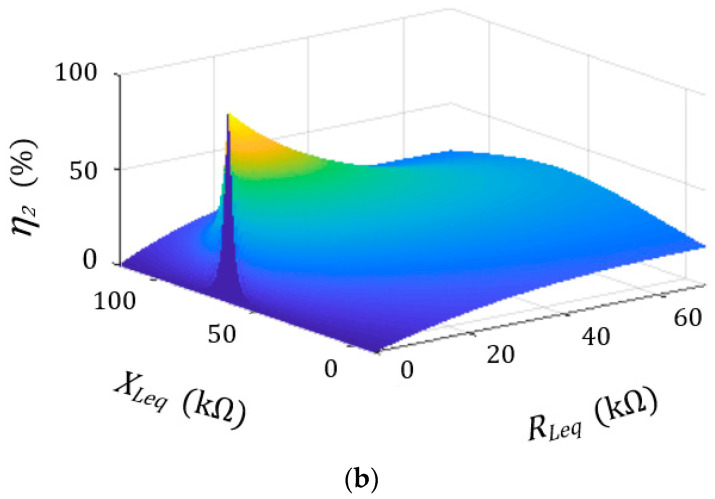
Efficiency comparison. (**a**) Original Mason circuit; (**b**) simplified circuit.

From the derivation of Mason equation, it was discovered that there exists a minor difference in the system performance between Equations (1) and (5). Observing the simulation verification in Figure 4, Figure 4a is Equation (1) and Figure 4b is Equation (5). The imaginary part and real part of the matching impedance will appear at the peak point of the figure. The value of imaginary part and the real part of the impedance matching of Equation (1) and simplified Equation (5) are almost identical, as is shown in Equation (6). In addition, it shows that impedance matching is indeed effective in improving system performance and can be calculated via circuit simplification, thereby reducing the amount of calculation.

As shown in Figure 3c, the matched impedance circuit configuration is a T network. Where the imaginary and real parts of the impedance matching are equal to the real and imaginary parts of $Z_{Leq}$, resulting in the peak point of the figure. Therefore, the partial derivatives of the real part $R_{Leq}$ and the imaginary part $X_{Leq}$ of $Z_{Leq}$ are calculated, respectively, based on Equation (5) to obtain the maximum efficiency value. Equation (7) is the partial derivatives of the real part $R_{Leq}$ of $Z_{Leq}$. Equation (8) is the partial derivatives of the imaginary part $X_{Leq}$ of $Z_{Leq}$. To simplify the lengthy formula, we replace the repeated equations within the procedure with variables A, B, C, D, E, F, and G, as indicated in Equation (9). Finally, the mathematical model is compared with the optimization module in MATLAB and found that the results are very close. The correctness of the mathematical model is proved. Additionally, it can be proved that the performance of the system can be improved through impedance matching.

(7) $$\begin{array}{l} \frac{\partial \eta }{\partial R_{\mathrm{leq}}}=\frac{I\sqrt{A}\sqrt{C}}{\sqrt{\left((-R_{21}^{2}-X_{21}^{2})(AR_{t1}+BR_{s1})C\right)}}\times (R_{21}^{2}R_{sw}^{2}R_{t1}+2R_{21}^{2}R_{sw}R_{t1}+R_{21}^{2}R_{tw}^{2}R_{t1}+X_{21}^{2}R_{sw}^{2}R_{t1}\\ +2R_{sw}R_{t1}R_{tw}X_{21}^{2}+R_{t1}R_{tw}^{2}X_{21}^{2}+R_{s1}R_{sw}^{2}X_{22}^{2}+2R_{s1}R_{sw}R_{tw}X_{22}^{2}+R_{s1}R_{{}_{tw}}^{2}X_{22}^{2}\\ -2R_{32}R_{s1}R_{sw}X_{22}X_{23}-2R_{32}R_{s1}R_{tw}X_{22}X_{23}+R_{{}_{32}}^{2}R_{s1}X_{{}_{23}}^{2}+X_{{}_{32}}^{2}R_{s1}X_{{}_{23}}^{2}+R_{23}^{2}R_{s1}C\\ -2X_{22}R_{s1}X_{leq}X_{32}X_{23}+R_{21}^{2}R_{t1}X_{leq}^{2}+X_{22}^{2}R_{s1}X_{leq}^{2}-2X_{22}R_{s1}X_{sw}X_{32}X_{23}+2R_{21}^{2}R_{t1}X_{leq}X_{sw}\\ +2X_{21}^{2}R_{t1}X_{leq}X_{sw}+2R_{s1}X_{22}^{2}X_{leq}X_{sw}+R_{21}^{2}R_{t1}X_{sw}^{2}+X_{21}^{2}R_{t1}X_{sw}^{2}+X_{22}^{2}R_{s1}X_{sw}^{2}\\ +2(ADR_{t1}+R_{s1}X_{22}(-X_{32}X_{23}+DX_{22}))X_{tw}+(AR_{t1}+R_{s1}X_{22}^{2})X_{tw}^{2}-2R_{22}R_{s1}X_{23}((-R_{sw}-R_{tw})\\ X_{32}+R_{32}E)-2R_{23}R_{s1}(R_{22}R_{32}F+FX_{22}X_{32}-X_{22}R_{32}E+R_{22}X_{32}E)+R_{22}^{2}R_{s1}(F^{2}+E^{2}){)}^{\left(\frac{1}{2}\right)} \end{array}$$

(8) $$\begin{array}{l} \frac{\partial \eta }{\partial X_{leq}}=\frac{1}{\left(R_{t1}A+R_{s1}B\right)}\times (R_{22}R_{32}R_{s1}X_{23}+X_{22}X_{32}R_{s1}X_{23}+R_{23}R_{s1}(R_{22}X_{32}-X_{22}R_{32})-R_{s1}R_{22}^{2}G\\ -R_{t1}AG-R_{s1}X_{22}^{2}G) \end{array}$$

(9) $$\begin{array}{l} A=R_{21}^{2}+X_{21}^{2}{\;B=R}_{22}^{2}+X_{22}^{2}\;F=R_{\mathrm{sw}}+R_{\mathrm{tw}}\\ {C=R}_{32}^{2}+X_{32}^{2}\;D=X_{\mathrm{leq}}+X_{\mathrm{sw}}\;G=X_{\mathrm{sw}}+X_{\mathrm{tw}}\\ E=X_{\mathrm{leq}}+X_{\mathrm{sw}}+X_{\mathrm{tw}}\; \end{array}$$

### 2.2. Proposed Approach for Impedance Matching—The Clamp of Piezoelectric Transducer

**Figure 5:**
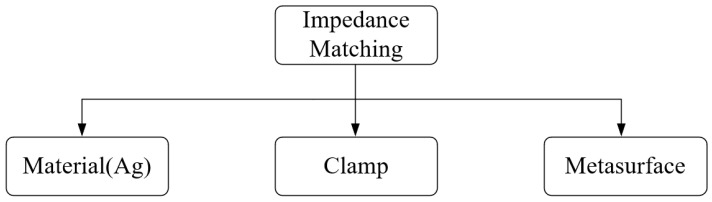
Method of impedance matching.

There are many methods of impedance matching [35,36,37], as shown in Figure 5. The method of traditional impedance matching uses special materials to coat the surface of the piezoelectric transducers, such as silver plating on the piezoelectric transducer, which has a low degree of freedom of operation, and it is difficult to control the thickness and uniformity of the application. A relatively new method of impedance matching is to use a meta-surface with a special structure to change the impedance [38]. However, the design and performance of an acoustic meta-surface are usually optimized for a specific application, so it is difficult to tune and change.

**Figure 6:**
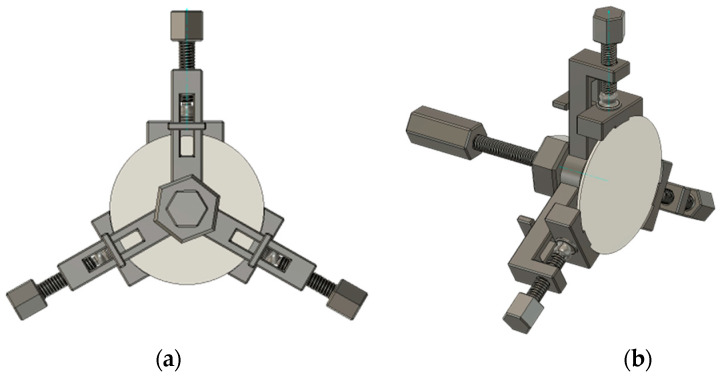
The model of the peripheral clamp. (**a**) Front view and (**b**) side view.

During this research, it was observed that the location of the fixed piezoelectric transducer influenced the vibration modes, which consequently impacted the impedance characteristics of the piezoelectric transducer. The traditional method is to fix the back of the piezoelectric transducer. Which is to glue it with the bracket to become a non-detachable, permanently bonded system. This method has many disadvantages, such as poor adjustability and stability. It was discovered that fixing the side of the piezoelectric transducer presents an effective means of adjusting impedance matching; however, this approach is scarcely mentioned in the existing literature. Even studies on detachable fixtures are focused on metals or solid media and mainly target the backside of the piezoelectric transducer, with no research on the side fixation, as is shown in Table 3. Therefore, this study demonstrates high innovation and practical applicability. A peripheral clamp consisting of three claws was proposed. The angle between each claw is 120 degrees, and the width of each claw corresponds to 30 degrees, as shown in Figure 6. This is a detachable clamp that can be adjusted for tightness, convenient, and flexible. A further advantage of the peripheral clamp is that they are all made using 3D printing, which reduces the price considerably.

## 3. Simulation and Experimental Verification

The finite element simulation software COMSOL Multiphysics was used in this paper, which is for experimental preparation and verification of the effectiveness of the novel clamp. COMSOL Multiphysics is a finite element simulation software for multiphysics coupling. It is based on the finite element method and realizes the simulation of natural physical phenomena by solving partial differential equations. It uses mathematical techniques to solve physical phenomena in the real world.

**Figure 7:**
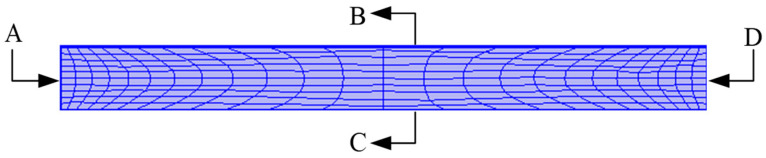
Mesh throwing of piezoelectric transducer in finite element simulation.

In order to reduce the calculation, a 2D axisymmetric model was used for the simulation. A model of the mesh is shown in Figure 7. A represents the axis of symmetry, B is the input voltage side, C is the ground side, and D represents the fixed constraint. Ultrasonic waves are generated by applying a voltage to a piezoelectric transducer. Therefore, the boundary conditions must be considered simultaneously, as the mechanical and electrical parts are related to the vibration applied to the piezoelectric transducer in the simulation. Table 4 lists the settings of its boundary conditions. In addition, we have not set up any additional air damping and only accounted for the losses in the materials.

### 3.1. The Clamp of Piezoelectric Transducers

As shown in Table 5, the commonly used physical quantity for sound measurement is sound pressure, but usually, the sound pressure level describes the magnitude of sound pressure. The conversion equation between sound pressure and sound pressure level is shown in Equation (10). The variation in the vibration amplitude determines the sound intensity. Sound intensity is calculated using energy and sound pressure when expressed by pressure. Sound intensity is a vector; sound pressure is a scalar. The relationship between sound intensity (*I*) and sound pressure (*p*) is shown in Equation (11), where *ρ* is medium density and *c* is sound velocity. Sound power refers to the total energy the sound source radiates to space per unit of time; the unit is W. The relationship between sound power and sound intensity is shown in Equation (12), where A is the area through which the sound wave passes vertically. This implies that the acoustic pressure becomes stronger as the sound pressure level increases. A stronger acoustic pressure corresponds to a higher sound intensity, and the acoustic power within a given area increases with greater sound intensity. With the increase in acoustic power, the energy becomes more pronounced, indicating the effectiveness of the clamp.

(10) $$L_{p}=20\times \log10\frac{p}{p_{0}}$$

(11) $$I=\frac{p^{2}}{\rho c}$$

(12) P=IA

The fundamental equations for piezoelectric transducers are shown in Equation (13), where *T* is the stress and *E* is the electric field, with both being independent variables, and where *c^D^*, *h*, and *β^S^* are the elastic stiffness coefficient, piezoelectric stiffness coefficient, and dielectric isolation rate, respectively. These equations state that the vibration strength and sound intensity increase with greater stress. Additionally, a higher sound intensity is indicative of a greater energy. Therefore, in COMSOL, we assess the effectiveness of different clamps by observing the sound pressure level and stress.

(13) $$\begin{array}{l} T=c^{D}S_{3}-hD_{3}\\ E=-hS_{3}+\beta^{S}D_{3} \end{array}$$

**Figure 8:**
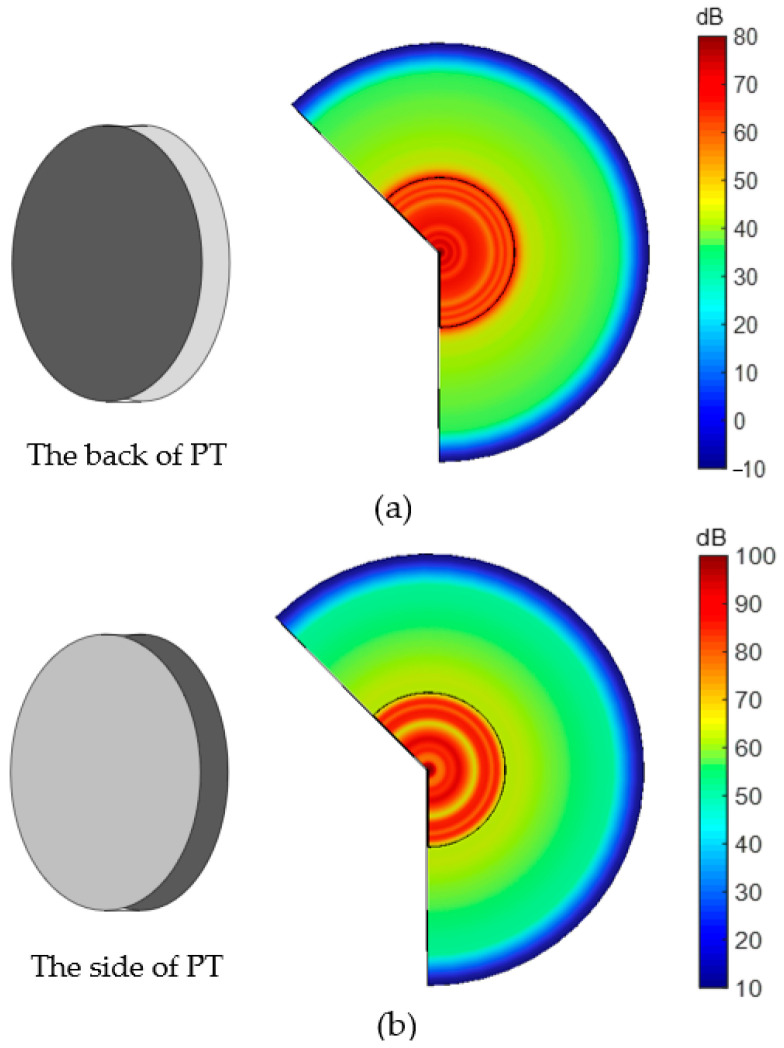
Total sound pressure level distribution of piezoelectric transducer. (**a**) Fixed piezo back; (**b**) fixed piezo side.

We used the acoustic–structure interaction module in the built-in acoustic module of COMSOL and added the Circuit module. The model of the APT system is established in COMSOL, the distance is about 2 mm, and the frequency is 51.5 kHz. As shown in Figure 8, the left is a schematic diagram, the dark colour is where the piezoelectric transducer is fixed, and the right is the sound pressure distribution obtained through COMSOL simulation. The dark part of Figure 8a is the back of the piezoelectric transducer. When the piezoelectric transducer is fixed on the back, the maximum sound pressure level of the total is 80 dB, which is considerably lower than that in Figure 8b. In the figure, the distribution in the middle is the sound pressure level on the piezoelectric transducers, and the surrounding distribution is the sound pressure level in air. The dark portion of Figure 8b represents the side of the piezoelectric transducer. When the piezoelectric transducer is fixed on the side, the maximum total sound pressure level is 100 dB, which is strong and uniformly distributed. This phenomenon shows that different fixed positions of piezoelectric transducers produce other effects. Setting the side of the piezoelectric transducer achieves a higher sound pressure than fixing the back.

#### 3.1.1. No Clamp

**Figure 9:**
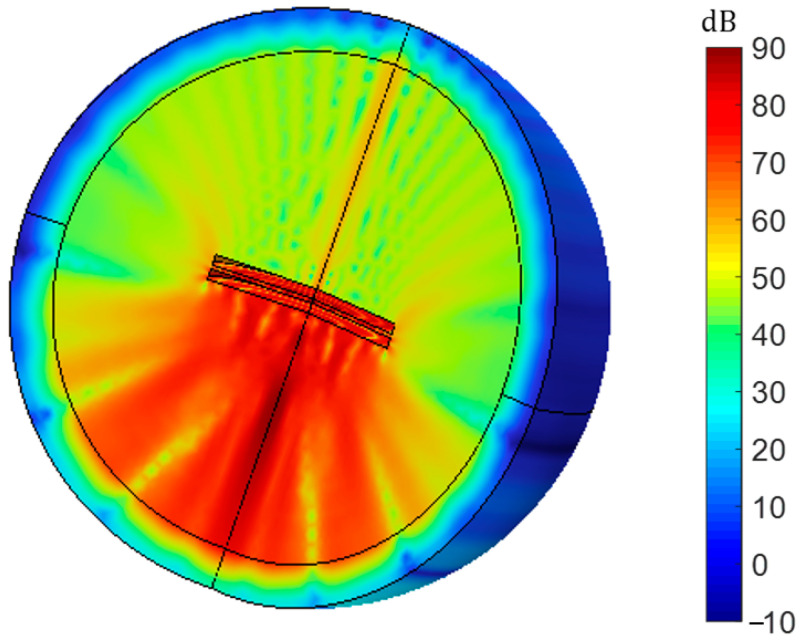
COMSOL model with no clamp and no fixed constraints.

The model of the piezoelectric transducer with a free state is built in COMSOL, as shown in Figure 9. It is the distribution of its total sound pressure level. In this context, we need to emphasize the “free state.” Due to the inability of the piezoelectric transducer to float in the air without any support, we applied fixed constraints in the COMSOL model. However, compared with the constraint areas on the back and sides mentioned in the paper, the constraint area in the no-clamp model is negligible. It is found that the energy of the transmitting piezoelectric transducer is almost diffused to the surroundings when the piezoelectric transducer is in a free state, and there is not much sound pressure transmitted to the receiver. This situation poses significant challenges to efficient energy transmission within the system. Therefore, the design of the fixture is very important.

#### 3.1.2. Back Clamp

**Figure 10:**
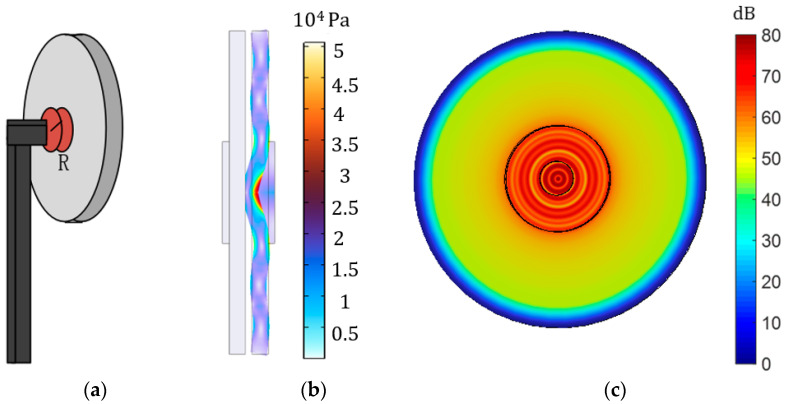
Fixed the back of piezo. (**a**) Schematic diagram of the back clamp; (**b**) the magnitude and deformation of the stress on the back clamp; (**c**) the distribution and size of the total sound pressure level on the back clamp.

The traditional method to fix a piezoelectric transducer is to glue it with the bracket to become a non-detachable, permanently bonded system. The holding clamp location is usually on the back of the piezoelectric transducer, as shown in Figure 10a. The model of the traditional clamp is established in COMSOL. The transmitter adds an input voltage of 8 V, and the output terminal is set to an open circuit state. The frequency is about 50 kHz. The distance between the transmitter and receiver is 2 mm. Figure 10b shows the stress transformation of the piezoelectric transducer, and its peak value is about 50 kPa. Figure 10c shows the change in its sound pressure level. The maximum value is about 80 dB. Compared with the free state, the stress has increased, but the peak value of the sound pressure level has not changed much. Nevertheless, this traditional clamp still has some influence on the distribution of sound pressure.

#### 3.1.3. Peripheral Clamp

**Figure 11:**
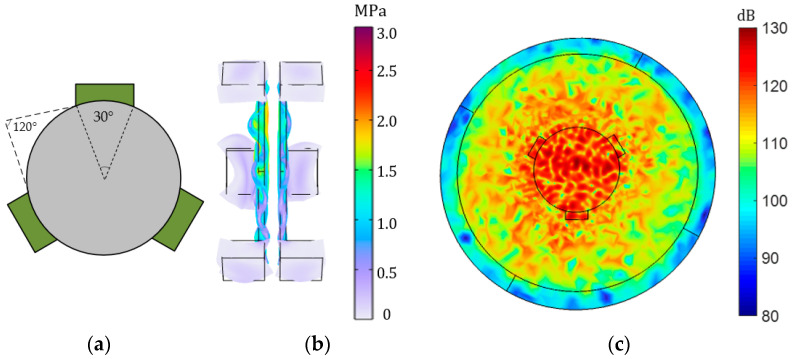
Fixed the side of piezo. (**a**) Schematic diagram of the peripheral clamp; (**b**) the magnitude and deformation of the side stress of the peripheral clamp; (**c**) the distribution and size of the total sound pressure level on the peripheral clamp.

A novel detachable clamp is designed in which the fixed position is on the side of the piezoelectric transducer, as shown in Figure 11a. The peripheral clamp has a total of three claws. The angle between each claw is 120 degrees, and the width of each claw corresponds to 30 degrees. The model of the peripheral clamp is established in COMSOL, and an input voltage of 8 V is added to the transmitter, and the output terminal is set to an open circuit state. The distance between the transmitter and receiver is 2 mm. The frequency is about 50 kHz. Figure 11b is the stress transformation of the piezoelectric transducer, and its peak value can reach 3 MPa. Figure 11c is the change in its sound pressure level; the maximum value can be up to 130 dB. The system performance is improved compared with the back clamp and no clamp. Noticeably, the sound pressure level has increased by nearly 50 dB.

#### 3.1.4. Piezoelectric Transducer Array

**Figure 12:**
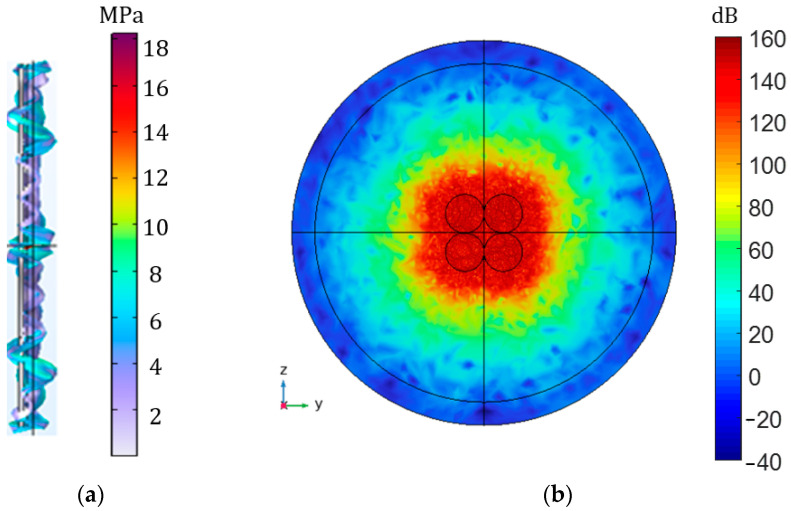
COMSOL model with PT array. (**a**) The magnitude and deformation of the stress of piezoelectric transducer array; (**b**) the distribution and size of the total sound pressure level of the piezoelectric transducer array.

Our current simulations and investigations are based on a single piezoelectric transducer, and the resulting values may be small compared with IPT. Additionally, because the acoustic impedance of the air does not match the acoustic impedance of the piezoelectric transducer seriously, its loss is very large. However, the research on APT systems in air is still very important, especially in biomedicine. For this reason, we made a simulation model of the PT array to prove that if the number of PTs increases, the result will obviously be improved. The model of the PT array is established in COMSOL, and the transmitter and receiver are composed of four piezoelectric transducers with a radius of 25 mm connected in parallel. The transmitter adds an input voltage of 8 V and sets the output to an open circuit state. The frequency is about 50 kHz. The distance between the transmitter and receiver is 2 mm. Figure 12a is the stress transformation of the piezoelectric array, and its peak value is up to 18 MPa. Figure 12b shows the change in its sound pressure level, with a maximum value up to 160 dB. The results can be nearly doubled compared with the results with no clamp. It proves that the number of PTs increases, and the experimental and simulation results will significantly improve.

**Figure 13:**
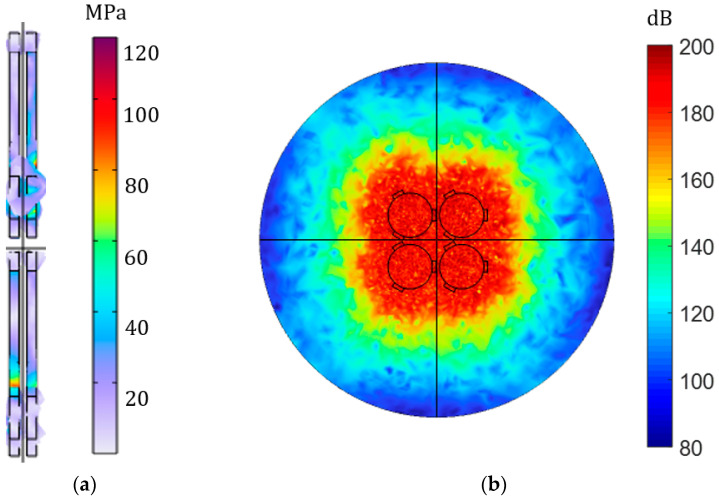
COMSOL model of a piezoelectric transducer array with peripheral clamp. (**a**) Magnitude and deformation of the piezoelectric transducer array with the peripheral clamp; (**b**) distribution and size of the total sound pressure level of the piezoelectric transducer array with the peripheral clamp.

Furthermore, the model in Figure 13 consists of piezoelectric transducer arrays with the same size and number of peripheral clamps. An input voltage of 8 V is applied to the transmitters, and the output is set to an open circuit state with a frequency of approximately 50 kHz. In Figure 13a, the stress transformation of the piezoelectric array with a peripheral clamp is presented with a peak value of up to 120 MPa. Figure 13b displays the change in sound pressure level, with a maximum value of 200 dB. These results are significantly higher than those of the piezoelectric array without a clamp, thus confirming the effectiveness of the clamp once again. 

### 3.2. Experimental Verification

**Figure 14:**
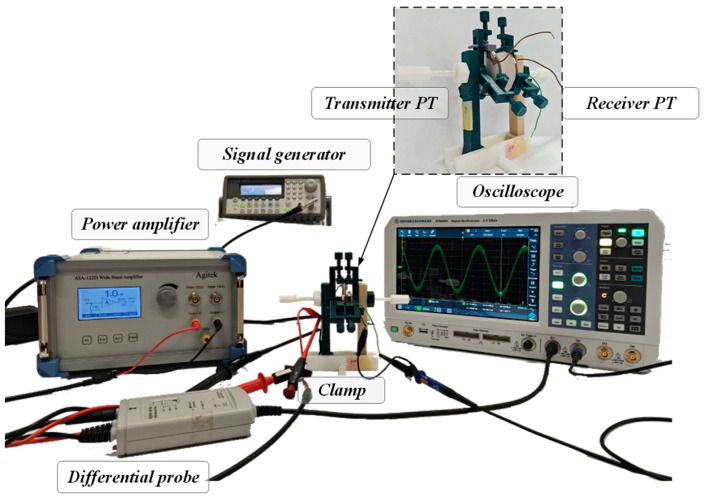
Experimental platform of APT system in air.

Under the condition that the resonant frequency of the system is 51.5 kHz, firstly, select the signal generator model Agilent 33250A and the ATA-122D Wide Band Amplifier as the system’s power supply; the receiving end circuit is an open circuit. The transmitting end uses a differential probe Agilent N2772A probe to detect waveform. The receiver uses an oscilloscope probe to observe the waveform. The size of the piezoelectric transducer is a circular ceramic sheet with a radius of 25 mm, and the piezoelectric material is PZT-4. The components of the clamp are all manufactured by a FLASHFORGE 3D printer, and the 3D material is PLA. An experimental diagram of the system is shown in Figure 14.

#### 3.2.1. Impedance Characteristics

**Figure 15:**
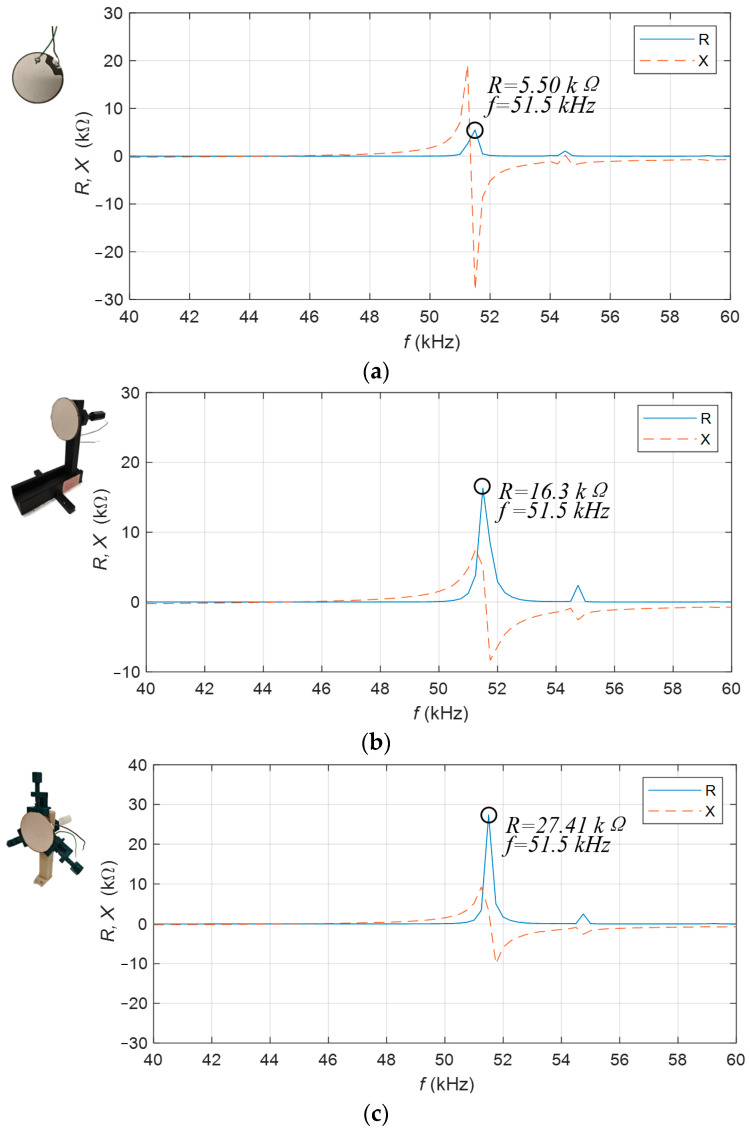
Impedance characteristics of a single piezoelectric transducer. (**a**) Without fixed constraints; (**b**) with fixed constraints on the back; (**c**) with fixed constraints on the sides.

The impedance characteristics of a single piezoelectric transducer are measured in different cases, including no clamp, with a back clamp, and with a peripheral clamp. Figure 15a shows the impedance characteristic of a single piezoelectric transducer without any constraints and clamp. Figure 15b is the impedance characteristic of conditions on the back with the back clamp of a single piezoelectric transducer. Figure 15c is the impedance characteristic of a single piezoelectric transducer with constraints on its sides with the peripheral clamp. The Agilent E4980A Precision LCR Meter is used to measure the impedance of the piezoelectric transducer. The resonant frequency of a single piezoelectric transducer is 51.5 kHz, and its real resistance is 5.5 kΩ. The resonance frequency of the piezoelectric transducer fixed on the back is 51.5 kHz, and its real resistance is 16.3 kΩ; The resonance frequency of the piezoelectric transducer fixed on the side is 51.5 kHz, and its real resistance is 27.41 kΩ. According to the measurement results, the piezoelectric transducer’s resistance is higher when using fixed constraints and a clamp, especially when the constraints with the peripheral clamp are added on the side. According to Equation (14), *P* is the sound pressure, *Z* is the acoustic impedance, and *c* is the volume velocity. When *c* is constant, the impedance is higher and the sound pressure is stronger.

(14) P=c Z

#### 3.2.2. Distance Characteristics

**Figure 16:**
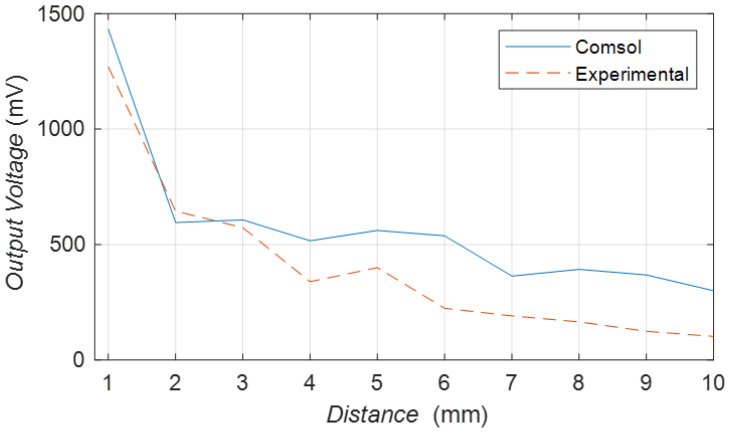
Simulation and experimental verification of the peripheral clamp.

As shown in Figure 16, the red line is the experimental data about the relationship between the output voltage and the distance between the transmitter and receiver. The blue line is the simulation data about the relationship between the output voltage and the distance between the transmitter and receiver. These data were obtained under uniform conditions. The transmitter adds an input voltage of 8 V and sets the output to an open circuit state. The frequency is about 50 kHz. The trends of these two sets of values are very similar, with decreasing output voltage as distance increases. This result validates the simulation model.

**Figure 17:**
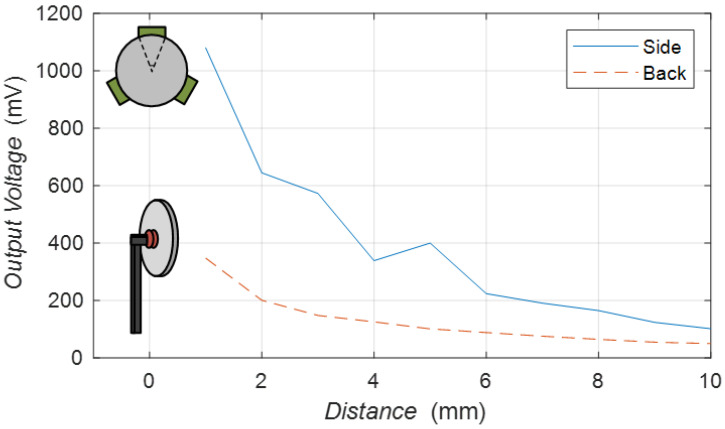
Distance characteristics of back clamp and peripheral clamp.

The experiment compared the relationship between different distances and the output voltage with two additional clamps, as shown in Figure 17. The clamp that fixed the piezoelectric transducer on the back is a cylinder with a radius of 8 mm. The clamp that set the side of the piezoelectric transducer is distributed in an equilateral triangle with a fixed angle of 30 degrees. The distance between the transmitter and receiver is from 1 mm to 10 mm. An input voltage of 8 V is applied to the transmitters, and the output is set to an open circuit state with a frequency of approximately 50 kHz.

The blue curve in Figure 17 is the peripheral clamp, and the red curve is the back clamp. The trend of all curves decreases with increasing distance. It can be found that the output voltage of the novel clamp is always higher than that of the fixed back clamp at different distances. Therefore, the novel peripheral clamp proposed in this paper is effective. 

## 4. Conclusions

In this paper, we introduced an impedance matching circuit to the Mason circuit and examined the correlation between input impedance and system efficiency. We identified a specific resistance value that maximizes efficiency, thus validating the efficacy of impedance matching. Furthermore, we compared different piezoelectric transducer configurations, including those with no fixed constraints, fixed constraints on the back, and fixed constraints on the side. Our findings revealed that the transducers with fixed constraints on the side exhibited higher sound pressure and output voltage compared with those without fixed constraints or with fixed constraints on the back. Based on these results, we proposed a novel peripheral clamp design, forming an equilateral triangle with a fixed width spanning 30 degrees at each point. This low-cost peripheral clamp can be entirely manufactured using 3D printers. We also investigated its impedance and distance characteristics, obtaining experimental results that aligned with our simulation outcomes, thus confirming the effectiveness of the novel peripheral clamp. While our studies primarily focused on a single piezoelectric transducer, we also conducted simulations involving piezoelectric arrays, demonstrating improved performance compared with performance with a single transducer. Therefore, if the measurement data from a single transducer are unsatisfactory, replacing it with a piezoelectric array can enhance the results.

## Figures and Tables

**Table 1:** Acoustic impendence.

Medium	Acoustic Impendence $Z_{0}=\rho c$	Density $\rho \;(kg/m^{3})$	Sound Speed c (m/s)
Steel	39 × 10^6^	7700	5000
Aluminium	14 × 10^6^	2700	5200
Water	1.44 × 10^6^	1000	1440
Air	410	1.2	340
PZT-5H	36 × 10^6^	7500	4800

**Table 2:** The parameters of piezoelectric components.

Material Properties		Symbol	Meaning
$k_{t}^{2}=\frac{e_{33}^{2}}{C_{33}^{D}\varepsilon_{33}^{s}}=\frac{h_{33}^{2}\varepsilon_{33}^{s}}{C_{33}^{D}}$	$\Gamma =\frac{\omega }{v_{D}}=\omega \sqrt{\frac{\rho }{C_{33}^{D}}}$	ρ	Density
		*t*	Thickness
$Z_{0}=\rho A\nu_{D}=A\sqrt{\rho C_{33}^{D}}$	$N=C_{0}h_{33}$	$\varepsilon_{33}^{s}$	Permittivity
$C_{0}=\frac{\varepsilon_{33}^{s}A}{t}$	$h_{33}=k_{t}\sqrt{\frac{C_{33}^{D}}{\varepsilon_{33}^{s}}}$	$k_{t}$	Electromechanical coupling
		*A*	Area
$Z_{s}=-jZ_{0}\csc\left(\Gamma t\right)$	$Z_{r}=jZ_{0}\tan\left(\frac{\Gamma t}{2}\right)$	$C_{33}^{D}$	Elastic stiffness

**Table 3:** APT models based on separating media and excitation methods.

Method	Ref		Medium	Power/Sound	Detachable
	Author	Year			
Special material	[9] Freychet 2020		Wall	Power	Non-detachable
	[10] Zhou 2022		Air	Power	Non-detachable
	[17] Rymantas 2017		Air	Sound	Detachable
Metasurface	[22] Eun 2018		Air/water	Sound	Detachable
Clamp	[25] Tseng 2022		Metal	Power	Detachable
This Research			Air	Power	Detachable

**Table 4:** The boundary state used for FEM model in simulation.

Boundary	Mechanical Status	Electrical Status
A	Axisymmetric	Axisymmetric
B	Free	Terminal Voltage
C	Free	Ground
D	Roller Support	Zero Charge

**Table 5:** Common physical quantities and their units.

Physical Quantity	Unit
*p* (Sound pressure)	Pa
*L_p_* (Sound pressure level)	dB
*I* (Sound intensity)	W/m^2^
*P* (Sound power)	W
*T* (Stress)	Pa

## Data Availability

Not applicable.
